# Rationale and concerns for using JAK inhibitors in axial spondyloarthritis

**DOI:** 10.1093/rap/rkae141

**Published:** 2024-11-07

**Authors:** Saad Ahmed, Rohan Yesudian, Hassan Ubaide, Laura C Coates

**Affiliations:** General Medicine, Colchester Hospital, Colchester, UK; School of Clinical Medicine, University of Cambridge, Cambridge, UK; General Medicine, Colchester Hospital, Colchester, UK; Nuffield Department of Orthopaedics, Rheumatology and Musculoskeletal Sciences, University of Oxford, Oxford, UK; Oxford NIHR Biomedical Research Centre, Oxford University Hospitals NHS Trust, Oxford, UK

**Keywords:** spondyloarthropathies, Janus kinase inhibitors, ankylosing spondylitis, axial spondyloarthritis management, non-radiographic axial spondyloarthritis, radiographic axial spondyloarthritis

## Abstract

Axial spondyloarthritis (axSpA) is a chronic illness with limited treatment options. The role of Janus kinase (JAK) inhibition as a therapeutic option has increasingly become a focus of research in recent years as they have brought a new mode of action to the clinical armamentarium. This review assesses the efficacy and safety profile of these drugs in axSpA. The current phase 2 and 3 clinical trials data are summarized across tofacitinib, upadacitinib and filgotinib. Moreover, the safety profiles of these drugs, in the context of emerging safety signals such as during the ORAL surveillance study, are reviewed. In summary, JAK inhibitors offer a novel therapeutic target for axSpA and appear to address some of the unmet needs for patients who have either failed to respond to current treatment options or in whom they are contraindicated. There is a relative lack of evidence in non-radiographic axSpA and longer-term trials are needed to establish true efficacy and safety profile in radiographic axSpA.

Key messages

- Janus kinase (JAK) is involved in facilitating cytokine signal transduction in the pathogenesis of spondyloarthropathies.
- JAK inhibitors could address an unmet need where other treatments have failed or are contraindicated.
- Longer-term trials are needed to establish efficacy and safety profiles in treating axSpA.

## Introduction

Axial spondyloarthritis (axSpA) is a form of chronic inflammatory arthritis characterized by inflammatory axial disease with sacroiliitis and enthesitis and less often peripheral arthritis. It is also associated with extra-articular associations including psoriasis, uveitis and inflammatory bowel disease (IBD) [1, 2]. Within the spectrum of axSpA, it is possible to define two groups: disease with radiographic (r-axSpA) SI joint changes defined by the modified New York criteria (traditionally referred to as AS) and disease without radiographic changes [non-radiographic (nr-AxSpA)] defined by clinical features including HLA-B27 positivity and evidence of sacroiliitis identified on MRI [1].

## Established axSpA treatment

The goal of treatment for patients with axSpA is to control inflammation, improve symptoms, maximize quality of life and prevent structural progression [3]. The Assessment of SpondyloArthritis international Society–European League Against Rheumatism (ASAS-EULAR) treatment recommendations cover the whole spectrum of axSpA. These recommendations state that NSAIDs and physiotherapy should be considered as first-line treatment [4]. There is a 70–80% short-term response rate for NSAIDs [5], although they are not sufficient to control disease in a substantial proportion of the axSpA population. Glucocorticoids and conventional synthetic DMARDs (csDMARDs) are considered ineffective in axial disease, unlike peripheral inflammatory arthritis [4]. However, sulfasalazine is only recommended to treat peripheral arthritis [6]. Methotrexate has been shown to be ineffective, although the quality of evidence for this is poor [7].

The 2022 update of the ASAS-EULAR recommendations for the management of axSpA [8] acknowledge the role of Janus kinase inhibitors (JAKis) alongside TNF inhibitors (TNFis) and IL-17 inhibitors (IL-17is). However, prior to this, the only treatments proven to treat axSpA were TNFis and IL-17is, which were recommended for patients with high disease activity despite NSAID use [8]. Most randomized controlled trials (RCTs) supporting these recommendations have recruited patients with AS or r-axSpA, although the guidelines recommend treatment with the same medications in nr-axSpA based on a smaller number of RCTs. Table 1 shows a summary of the clinical trials program for established treatments in axSpA.

**Table 1.** Summary of clinical trial data for established treatments in r-axSpA and nr-axSpA

Reference	Study type	Arms	Primary endpoint	Results	Data for radiographic change
van der Heijde *et al*. [9]	Phase 3 RCT	Adalimumab 40 mg Q2W *vs* PBO	ASAS20 at week 12	Adalimumab superior to PBO: 40 mg 58.5%, PBO 20.5%	No data
Haibel *et al*. [10]	RCT	Adalimumab 40 mg Q2W, PBO	ASAS20 at week 12	Adalimumab is superior to PBO: adalimumab 40 mg 68.2%, PBO 25%	No data
RAPID-axSpA study, Landewé *et al*. [11]	Phase 3 RCT	Certolizumab 200 mg Q2W *vs* 400 mg Q4W *vs* PBO	ASAS20 at week 12	Certolizumab superior to PBO: 200 mg Q2W 57.7%, 400 mg Q4W 63.6%, PBO 38.3%	No radiographic data, only BASMI assessed
van der Heijde *et al*. [12]	RCT	Etanercept 25 mg twice weekly, etanercept 50 mg weekly, PBO	ASAS20 at week 12	Etanercept superior to PBO: etanercept 25 mg twice weekly 71.3%, etanercept 50 mg once weekly 74.2%, PBO 37.3%	No radiographic data
EMBARK, Maksymowych *et al*. [13]	Phase 3 RCT	Etanercept 50 mg once weekly, PBO	ASAS40 at week 12	Etanercept is superior to PBO: etanercept 33%, PBO 14%	Significant improvement SPARCC SI joint and spinal MRI scores week 12 and week 48
Braun *et al*. [14]	RCT	Infliximab 5 mg/kg, PBO	Disease regression by half at week 12	Infliximab is superior to PBO: infliximab 5 mg/kg 53%, PBO 9%	No radiographic data, only BASMI assessed
GO-RAISE, Inman *et al*. [15]	Phase 3 RCT	Golimumab 50 mg Q2W, golimumab 100 mg Q4W, PBO	ASAS20 at week 14	Golimumab is superior to PBO: golimumab 50 mg Q2W 59.4%, golimumab 100 mg Q4W 60.0%, PBO 21.8%	No radiographic data
Sieper *et al*. [16]	Phase 3 RCT	Golimumab 50 mg Q4W, PBO	ASAS20 at week 16	Golimumab is superior to PBO: golimumab 71.1%, PBO 40.0%	Significant improvement in SPARCC and MRI scores for sacroiliitis
MEASURE 1 and MEASURE 2, Baeten *et al*. [17]	Phase 3 RCTs ×2	MEASURE 1: secukinumab i.v. load + 75 mg monthly, secukinumab i.v. load + 150 mg monthly, PBO MEASURE 2: secukinumab s.c. load + 75 mg monthly, secukinumab s.c. load + 150 mg monthly, PBO	ASAS20 at weeks 16 and 52	Secukinumab is superior to PBO: MEASURE 1 (i.v. secukinumab): 75 mg secukinumab 60% (16 weeks) and 62% (52 weeks), 150 mg secukinumab 61% (16 weeks) and 63% (52 weeks), PBO 29% (16 weeks) MEASURE 2 (s.c. secukinumab): 75 mg secukinumab 41% (16 weeks) and 53% (52 weeks), 150 mg secukinumab 61% (16 weeks) and 63% (52 weeks), PBO 28% (16 weeks)	No data
MEASURE 3, Pavelka *et al*. [18]	Phase 3 RCT	Secukinumab i.v. load + 300 mg monthly, secukinumab i.v. load + 150 mg monthly, PBO	ASAS20 at week 16 and 52	Secukinumab is superior to PBO: 300 mg secukinumab 60.5% (16 weeks) and 68.4% (52 weeks), 150 mg secukinumab 58.1% (16 weeks) and 58.1% (52 weeks), PBO 36.8% (16 weeks)	No data
MEASURE 4, Kivitz *et al*. [19]	Phase 3 RCT	Secukinumab 150 mg s.c. load, secukinumab 150 mg s.c. non-loading dose, PBO	ASAS20 at weeks 16 and 104	Secukinumab, both in loading and non-loading regimens, is superior to PBO: loading dose 59.5% (16 weeks) and 74.0% (104 weeks), non-loading dose 61.5% (16 weeks) and 77.5% (104 weeks), PBO 47.0% (16 weeks)	No data
Deodhar *et al*. [20]	Phase 3 RCT	Secukinumab loading dose, secukinumab non-loading, PBO	ASAS40 at week 16 (loading dose), ASAS40 at week 52 (non-loading)	Both loading and non-loading secukinumab are superior to PBO: loading dose 41.5%, non-loading dose 39.8%, PBO 29.2%	Improvement in SI joint oedema
SURPASS, Baraliakos *et al*. [21]	Phase 3b RCT	Secukinumab 150 mg, secukinumab 300 mg, adalimumab 40 mg	Proportion of patients with no radiographic progression, measured by the modified Stoke Ankylosing Spondylitis score at week 104	No difference in rates of radiographic progression between secukinumab and adalimumab: secukinumab 150 mg 66.1%, secukinumab 300 mg 66.9%, adalimumab 40 mg 65.6%	Reduction in radiographic progression for both secukinumab and adalimumab up to 104 weeks
van der Heijde *et al*. [22]	Phase 3 RCT	Ixekizumab 80 mg Q2W, ixekizumab 80 mg monthly, adalimumab 40 mg Q2W, PBO	ASAS40 at week 16	Ixekizumab Q2W is superior to Q4W regimen, adalimumab 40 mg Q2W and PBO: ixekizumab 80 mg Q2W 52%, ixekizumab 80 mg monthly 48%, adalimumab 40 mg Q2W 36%, PBO 18%	Significant improvements in MRI SPARCC SI joint scores
COAST-V and COAST-W, Dougados *et al*. [23]	Two phase 3 RCTs: COAST-V bDMARD naïve, COAST-W TNFi experienced	COAST-V: ixekizumab 80 mg Q2W, ixekizumab 80 mg Q4W COAST-W: ixekizumab 80 mg Q2W, ixekizumab 80 mg Q4W	ASAS40 at week 52	COAST-V: ixekizumab 80 mg Q2W 50.6%, ixekizumab 80 mg Q4W 53.1% COAST-W: ixekizumab 80 mg Q2W 30.6%, ixekizumab 80 mg Q4W 34.2%	Sustained significant improvements in MRI SPARCC SI joint scores for 52 weeks
Deodhar *et al*. [24]	Phase 3 RCT	Ixekizumab 80 mg Q2W, ixekizumab 80 mg monthly, PBO	ASAS40 at week 16	Both ixekizumab 80 mg Q2W and monthly regimens are superior to PBO: ixekizumab Q2W 30.6%, ixekizumab 80 mg monthly 25.4%, PBO 12.5%	Significant improvements in spinal MRI SPARCC SI joint scores
COAST-X, Deodhar *et al*. [25]	Phase 3 RCT	Ixekizumab 80 mg monthly, ixekizumab 80 mg Q2W, PBO	ASAS40 at weeks 16 and 52	Ixekizumab is superior to PBO: ixekizumab 80 mg monthly 35% (week 16), 30% (week 52), ixekizumab 80 mg Q2W 40% (week 16), 31% (week 52), PBO 19% (week 16) and 13% (week 52)*	Significant reductions in SPARCC SI joint scores
van der Heijde *et al*. [26]	Phase 3 RCT	Bimekizumab 160 mg monthly, PBO	ASAS40 at week 16	Bimekizumab is superior to PBO: bimekizumab 160 mg monthly 44.8%, PBO 22.5%	Significant reductions in mean MRI Berlin spine and SPARCC SI joint scores
van der Heijde *et al*. [27]	Phase 2b trial	Bimekizumab 16 mg monthly, bimekizumab 64 mg monthly, bimekizumab 160 mg monthly, bimekizumab 320 mg monthly, PBO	ASAS40 at week 12	Bimekizumab is superior to PBO: bimekizumab 16 mg monthly 29.5%, bimekizumab 64 mg monthly 42.6%, bimekizumab 160 mg monthly 46.7%, bimekizumab 320 mg monthly 45.9%, PBO 13.3%	MRI performed in a subset of patients. Reductions in SPARCC SI joint scores were observed in all bimekizumab groups. Reductions in Berlin spine scores were observed in the three highest bimekizumab dose groups
Erdes *et al*. [28]	Phase 2 RCT	Netakimab 40 mg Q2W, netakimab 80 mg Q2W, netakimab 120 mg Q2W, PBO	ASAS20 at week 16	Netakimab is superior to PBO: netakimab 40 mg Q2W 72.7%, netakimab 80 mg Q2W 81.8%, netakimab 120 mg Q2W 86.9%, PBO 42.9%	No data
Wei *et al*. [29]	Phase 3 RCT	Brodalumab 210 mg, PBO	ASAS40 at week 16	Brodalumab is superior to PBO: brodalumab 210 mg 43.8%, PBO 24.1%	No data

Q2W: every 2 weeks; Q4W: every 4 weeks.

TNFis have been a mainstay for axSpA treatment, with five drugs available internationally. The ATLAS trial (NCT00085644), a multicentre, randomized, double-blind, PBO (placebo)-controlled trial, found a ≥20% response (ASAS20) at week 12 was achieved in >58.2% of participants receiving adalimumab *vs* 20.6% of subjects in the PBO arm. This response was sustained through week 24 for the adalimumab patients [9]. Infliximab was shown to be effective by the ASSERT trial (NCT00207701), which found that after 24 weeks, 61.2% of patients in the infliximab group were ASAS20 responders compared with 19.2% of patients in the PBO group (*P* < 0.001) [30]. The ASCEND phase 3 trial (NCT00247962) tested the efficacy of etanercept *vs* sulfasalazine and found the proportion of ASAS20 responders at week 16 was greater among patients treated with etanercept compared with those treated with sulfasalazine (75.9% *vs* 52.9%; *P* < 0.0001) [31]. In GO-RAISE (NCT00265083), a phase 3, PBO-controlled trial of golimumab, 78 patients were assigned to the 50-mg, 100-mg and PBO groups, respectively. After 14 weeks, 59.4%, 60.0% and 21.8% of patients, respectively, were ASAS20 responders (*P* < 0.001). A 40% improvement in the ASAS criteria at week 24 occurred in 43.5%, 54.3% and 15.4% of patients, respectively [15].

IL-17is have also demonstrated efficacy in axSpA. Two phase 3, double-blind trials, entitled MEASURE 1 (NCT01358175) and MEASURE 2 (NCT01649375) investigated subcutaneous and intravenous administration of secukinumab, respectively [17]. In MEASURE 1, the ASAS20 response rates at week 16 were 61%, 60% and 29% for subcutaneous secukinumab at doses of 150 mg and 75 mg and for PBO, respectively (*P* < 0.001 for both comparisons with PBO); in MEASURE 2, the rates were 61%, 41% and 28% for subcutaneous secukinumab at doses of 150 mg and 75 mg and for PBO, respectively (*P* < 0.001 for the 150-mg dose and *P* = 0.10 for the 75-mg dose). The significant improvements were sustained through 52 weeks [17]. Ixekizumab was investigated in a phase 3, double-blind RCT in TNFi-naïve r-axSpA patients and 16-week ASAS40 responses were observed in 51.8% of those receiving ixekizumab every 2 weeks, in 48.1% of those receiving ixekizumab every 4 weeks and in 18.6% of those receiving PBO [22].

Although with less data, prior RCTs have confirmed the efficacy of TNFis in nr-axSpA. There is some variability in the magnitude of response within this RCT data, likely due to differing inclusion criteria [32]. The RAPID-ax-SpA trial (NCT01087762) was a phase 3, double-blind RCT involving 325 patients with axSpA, 147 of whom had non-radiographic disease, that looked at the efficacy of certolizumab after 24 weeks [33]. A total of 62.7% achieved ASAS20 at week 12 (for the 400 mg every 4 weeks dosage) compared with 40% in the treatment and PBO arms, respectively. Four-year imaging outcomes were further assessed in certolizumab-treated patients and showed maintenance in the decrease of spinal and SI joint MRI inflammation up to week 204. A decrease in structural damage progression was observed with long-term certolizumab therapy, with 80.6% of AS patients meeting this outcome and therefore maintaining their modified Stoke Ankylosing Spondylitis spine score to within 2 units of their baseline.

A double-blind RCT evaluating the efficacy and safety of adalimumab in nr-axSpA patients, with a 52-week open-label extension, resulted in 54.5% of patients achieving an ASAS40 response compared with 12.5% in the PBO group (*P* = 0.004) [10]. In addition, ASAS partial remission was achieved in 22.7% and 0% in the treatment and PBO arms, respectively, at week 12. Predictors of an ASAS40 response included young age and elevated CRP. A further RCT looking at adalimumab in active nr-axSpA revealed similar results with 36% *vs* 15% achieving ASAS40 at week 12 in the treatment and PBO arms, respectively (*P* < 0.001) [34].

Etanercept was licensed by the European Medicines Agency in June 2014 after completion of the phase 3 EMBARK trial (NCT02319837) [13]. In this trial, patients with nr-axSpA who fulfilled ASAS criteria with symptom duration up to 5 years were recruited. A total of 32% of patients from the etanercept arm *vs* 16% of PBO patients achieved ASAS40 response at week 12. A better clinical response to etanercept showed an association with decreased CRP levels and bone marrow oedema on MRI of the SI joints.

Efficacy and safety data from the ESTHER trial (NCT00844142), which included r-axSpA and nr-axSpA patients treated with etanercept *vs* sulfasalazine, were similar for both patient groups and a sustained and consistent 4-year response was shown [35]. Data showed active MRI inflammation demonstrated significant improvement in active axial lesions and a correlation with good clinical response [35]. Similar results in efficacy and safety were seen in both subgroups, r-axSpA and nr-axSpA, suggesting etanercept’s treatment response is the same across the disease spectrum given a similar baseline level of inflammation. ASAS40 response was also seen in 70% of etanercept-treated patients compared with 31% in the sulfasalazine group.

Golimumab was also studied in the phase 3 double-blind RCT GO-AHEAD (NCT01453725), which looked at patients with symptom duration up to 5 years and non-radiographic disease compared with PBO until week 16, with an open-label extension until week 60 [16]. ASAS20 response at week 16 was the primary endpoint, with 71.1% and 40% achieving this in the treatment and PBO groups, respectively (*P* < 0.001). No difference in response was observed in patients with a baseline normal CRP and negative MRI for inflammation. In addition, an ASAS40 response, a secondary endpoint, was achieved in 56.7% *vs* 23% in the two patient groups (*P* < 0.0001). Golimumab was shown to be well tolerated with a favourable benefit–risk ratio and showed a greater improvement in symptoms in patients with active nr-axSpA compared with PBO.

In addition to the data for TNFis, data for nr-axSpA also exists for the IL-17is secukinumab and ixekizumab. The COAST-X study (NCT02757352) showed ixekizumab administered every 2 weeks achieved ASAS40 in 31% of patients compared with 13% of the PBO group at 52 weeks [25]. The PREVENT study (NCT02696031) was the first phase 3 RCT to assess the efficacy and safety of secukinumab in TNF-naïve nr-axSpA patients followed for up to 52 weeks, and included treatment groups receiving secukinumab with or without an initial loading dose [20]. A significantly higher proportion of patients achieved an ASAS40 in both treatment arms, 41.5% and 39.8%, compared with PBO at 19.9% at 52 weeks, demonstrating significant and sustained improvements.

Bimekizumab, a dual IL-17A and IL-17F inhibitor, was assessed in patients who had an inadequate response to NSAIDs therapy through a parallel, phase 3, randomized, double-blind, PBO-controlled trial [26]. Patients with r-axSpA were randomized in a 2:1 ratio to receive subcutaneous 160 mg bimekizumab every 4 weeks (Q4W) or PBO. The primary outcome measured the achievement of ASAS40 at 16 weeks, yet follow-up continued to 52 weeks. Significant results were observed at week 16, with 45% of bimekizumab participants achieving ASAS40 compared with 23% in the PBO group. Furthermore, significant reductions in the MRI Spondyloarthritis Research Consortium of Canada (SPARCC) SI joint inflammation scores were observed in the bimekizumab population, indicating objective radiographic improvement. No new safety issues were identified. Rapid responses to medication were seen, with a significant proportion of participants reaching ASAS40 from week 1 in both the preliminary phase 2b and subsequent phase 3 trials [26, 27]. This positions bimekizumab as an effective long-term treatment option for r-axSpA patients who do not respond adequately to NSAIDs.

Recent phase 3 data support the efficacy of bimekizumab treating both nr-axSpA [BE MOBILE 1 (NCT03928704)] and r-axSpA [BE MOBILE 2 (NCT03928743)] [26]. These parallel 52-week trials included patients with r-AxSpA and nr-AxSpA treated with bimekizumab 160 mg monthly compared with PBO. The primary endpoint was ASAS40 at week 16. In the nr-axSpA cohort, 47.7% of patients treated with bimekizumab achieved an ASAS40 at week 12 compared with 21.4% in the PBO arm, with similar responses observed in both TNF-naïve and TNF-inadequate responders. Improvements were also seen in objective inflammatory measures such as CRP, MRI lesions and Axial Spondyloarthritis Disease Activity Scores (ASDASs) also showed an improvement. No new safety concerns were identified. This study shows that dual inhibition of both IL-17A and IL-17F leads to rapid and sustained clinical improvements in r-axSpA and nr-axSpA patients compared with PBO.

Encouraging phase 2 and phase 3 results are seen for netakimab and brodalumab [28, 29], IL-17A and IL-17A receptor inhibitors, respectively. These drugs are licensed in Russia and Japan, respectively. Trials are ongoing for both agents. Data for brodalumab for 68-weeks show sustained efficacy and a good safety profile in axSpA patients [36]. ASAS responses were similar to those of other trials, but the generalizability of results is challenging due to the Asian ethnicity of the study population, small sample size and the open-label trial design. A multicentre phase 3 trial of netakimab *vs* placebo in r-axSpA patients showed ASAS40 response rates of 40.4% *vs* 2.6% at week 16, respectively [37]. These results are promising, but further phase 3 data are needed prior to positioning these drugs alongside other licensed IL-17is in axSpA. The novel tyrosine kinase (TYK) 2 inhibitor deucravacitinib, whose allosteric inhibitory mechanism of action affects both adaptive and innate immune responses [38], is currently undergoing phase 3 trials in PsA after encouraging a 20% improvement in ACR responses from its phase 2 program [39]. It is important to note, however, that the aforementioned data are from PsA studies with nothing currently available for axSpA.

Despite the availability of the current biologic DMARDs (bDMARDs) for axSpA, many patients fail to achieve or maintain low disease activity [40, 41]. Potential explanations for this may be altered drug metabolism, non-compliance, drug pharmacodynamics and immunogenicity, among others. Gender, smoking and obesity are also important factors influencing drug survival [42–44].

When faced with treatment failure, switching to another mode of action or cycling within the same mode should be discussed. Both TNFi and IL-17i have a good safety profile and reduce disease activity, but data for superiority of either of these classes over the other does not currently exist.

## Unmet need for additional treatments in axSpA

The advent of biologics has revolutionized the management of axSpA with effective therapies to control spinal inflammation. However, some people do not respond to TNFis or IL-17is and these were the only two mode of action drugs available to treat axSpA.

Response rates to TNFis and IL-17is remain modest, with only 60–65% of patients achieving a clinical response after a first bDMARD [45]. There are also contraindications to TNFis and IL-17is that may limit choices for particular patients (e.g. the presence of active IBD that may mean that IL-17is are not appropriate). Both medications require administration via the parenteral route, with no oral options available.

axSpA is a lifelong condition. While people may respond to initial treatment with TNFis or IL-17is, often the response can be lost over time. Long-term response is limited, with 50–60% of patients discontinuing TNFis within 2 years [46]. This so-called secondary non-response is a significant issue for a lifelong disease that often presents in the second or third decade of life. There is an unmet need for additional therapies that can adequately control inflammation in the spine. Limited data exist for switching strategies, with no RCT evidence for TNFi efficacy for patients with inadequate response to previous TNFis [47]. There is some evidence for secukinumab in TNF in adequate responders, albeit less than for TNF-naïve patients [48].

Although the number of biologics available in rheumatology has expanded rapidly in recent years, several therapies used in RA and PsA have not shown efficacy in axial disease. Tocilizumab (anti-IL-6) failed to achieve the desired endpoint for demonstrating efficacy [49]. IL-1 antagonism with anakinra was tested and improved spinal symptoms in only a small subgroup of patients with no significant response [50]. B cell inhibition with rituximab was tested in a phase 2 clinical trial on axSpA patients in whom TNFi had failed or not been tried. Efficacy was only demonstrated in TNFi-naïve patients, with no significant effect on ASAS20 responses in patient who did not respond to TNFi [51]. Therapies targeting IL-23, including ustekinumab (a p40 IL-12/23 inhibitor) and drugs targeting only IL-23 (p19 inhibitors), have shown efficacy in psoriasis and peripheral PsA trials, but negative trials in r-axSpA [52].

The role of IL-23 in the development of axSpA and as a treatment target is under discussion. IL-23 receptor polymorphism may influence both the disease phenotype and treatment response [53]. Potential reasons for the lack of efficacy of IL-23 inhibition in axSpA may relate to entheseal cells lacking the IL-23 receptor, IL-23 itself not being significantly involved in ongoing inflammation and IL-23-independent communication between immune and stromal cells [54]. Further elements to the research agenda in axSpA include assessing the impact of comorbidities on treatment response, assessing disease activity with concomitant fibromyalgia and the management of residual pain. The efficacy and safety of combined bDMARD and targeted synthetic DMARD therapy is also unclear, as is the impact of tapering therapy on structural damage progression [55]. The concept of combined clinical and imaging remission is also being studied in axSpA, with some data suggesting such a deeper sustained remission may bring improvements in functional attainment [56, 57].

The pleiotropic nature of cytokines means that non-response to a single anti-cytokine agent does not preclude that cytokine from having a role in disease pathogenesis. Targeting a combination of cytokines that may have failed as individual targets is still worthwhile, and this is where JAK inhibition comes in, blocking receptors of numerous cytokines and their downstream effects [58]. JAK inhibitors (JAKis) are an oral medication with a rapid onset of action that have demonstrated efficacy across multiple domains of axSpA and have become an integral component of national/international guidelines [8, 59]. The factors that may influence drug choice for a given axSpA patient may include clinician familiarity, the presence of extra-articular manifestations (EAMs) and relevant comorbidities, cost, safety and the risk of radiographic progression.

## JAKis in axSpA

### JAK inhibition

The newest drugs with proven efficacy in axSpA are the JAKis. In 2020, the EULAR released a consensus statement on the role of JAKis in immune-mediated inflammatory diseases [60]. This described how the phase 2, 12-week data available at that time demonstrated similar efficacy of JAKis compared with TNFis in r-axSpA and other inflammatory arthritides.

Broad direct inhibitory activity has been shown with various JAKs, such as upadacitinib, which blocks JAK1-dependent pathways such as IFN-γ, IL-6, IL-2 and IL-5 pathways, but also indirectly blocks JAK1-independent pathways such as IL-1, IL-23, IL-17 and IL-18, reducing leucocyte activation and inflammatory responses [61, 62]. Preclinical work has shown the benefits of JAK/signal transducer and activator of transcription (STAT) blockade in SpA, including halting the development of enthesitis and TNF-independent mechanisms [63, 64], which laid the groundwork for further trials.

In 2022 they were included in the ASAS-EULAR axSpA recommendations update. Recommendation 9 advises that they can be used in patients with persistently high disease activity despite conventional treatment. However, the recommendation emphasizes current practice, which is to use TNFis/IL-17is before JAKis. This is due to the longer clinical experience with these drugs, a larger evidence base and more long-term drug safety knowledge. There is also a paucity of evidence looking at the efficacy of JAKis in nr-axSpA. The JAKis are a novel group of highly effective agents that have shown efficacy in trials of r-axSpA and that now have the potential to address some of the unmet need in axSpA. They also provide the first effective oral therapy licensed for the treatment of axSpA. However, there have also been some safety concerns raised with this mode of action. This review assesses the efficacy and safety profile of JAKis in axSpA.

### JAK-STAT pathway

**Figure 1.**
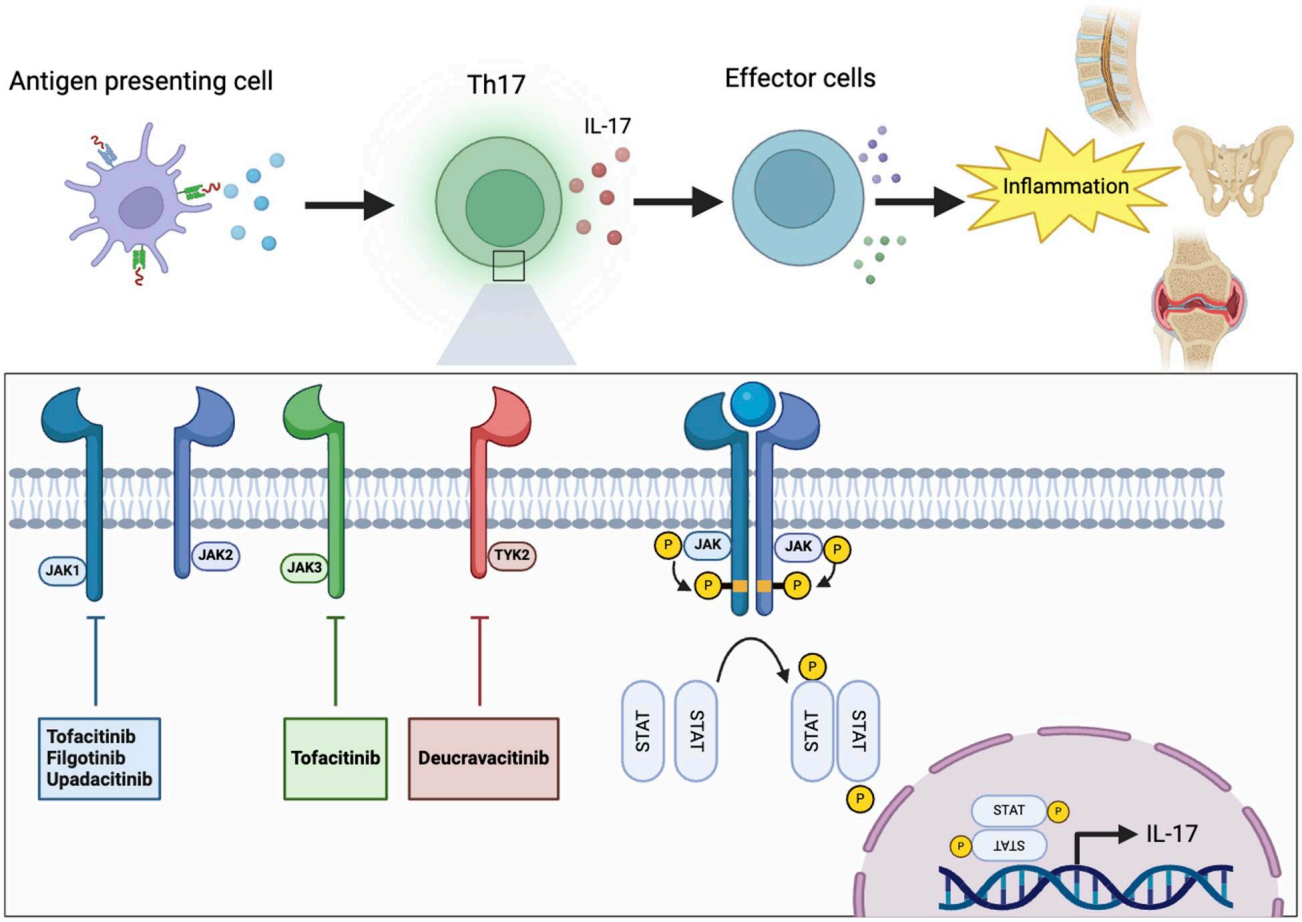
Pathogenesis of axSpA and molecular targets of JAK/STAT signalling

The inflammatory processes of axSpA are complex (see Fig. 1). Pro-inflammatory cytokines use signal induction pathways mediated by JAK. The JAK/STAT pathway facilitates the transduction of many cytokines and other molecules and has a pivotal role in many immune-related conditions [65]. There are four members of the JAK family: JAK1, 2 and 3 and TYK2. They are all cytoplasmic tyrosine kinases whose role is to phosphorylate tyrosine residues on themselves or other molecules, such as STATs. Type 1 and type 2 receptors are part of this effector pathway and other molecules such as ILs, IFNs, colony stimulating factors and hormones function via the JAK/STAT pathway [66]. JAKs contain receptor subunits and these may be associated with more than one JAK, thus more than one JAK pathway may be inhibited by blocking these molecules, providing a potential explanation for their variable efficacy and safety profiles [58]. The rapid clinical efficacy, oral formulation, shorter half-life relative to bDMARDs and lack of immunogenicity make JAK inhibition an exciting therapeutic target [67].

JAKis influence several of the key cytokines involved in the IL-23/IL-17 axis, a pivotal pathway in SpA [68]. The JAK/TYK2 combination supports the signalling of IL-23; blocking this also leads to downstream blockade of IL-17 production [69]. TNF production by macrophages, mediated by the Th1 cell response, is driven by IFN-γ and IL-12 signalling, which are supported by JAK1/JAK2 and JAK2/TYK2, respectively [2]. Therefore, even though TNF is not directly affected by JAK inhibition, blockade of the aforementioned JAKs will modulate its production. IL-17A is produced by CD4^+^ Th17 cells, the differentiation of which is governed by IL-23, which signals through JAK2/TYK2, and this further supports the downstream inhibition of IL-17 production through JAK inhibition [70].

### Key trials in axSpA

There are several trials of different JAKis in r-axSpA that have resulted in licenses for some of the drugs reviewed. Tofacitinib is a potent JAK1 and JAK3 inhibitor. The first study, a phase 2 randomized, placebo-controlled trial assessed the efficacy and safety of tofacitinib in r-axSpA patients with active disease [71]. This study used an Emax model to examine the dose–response relationship for ASAS20 response rates. The model predicted ASAS20 response rates of 63% and 67.4%, respectively, for the 5 mg and 10 mg twice daily dose of tofacitinib at week 12. However, the 5 mg twice daily dose was the only dose statistically superior to placebo at week 12 for ASAS20 response; sampling variability may account for this. The 10 mg dose was numerically superior to placebo, but this difference was not statistically significant. In addition, patient-reported outcomes and other measures of disability, such as the European Quality of Life 5-Dimensions questionnaire, 36-item Short Form Health Survey (SF-36) and AS Quality of Life (ASQoL), and work impairment were shown to be improved in all treatment arms, although not in a dose-dependent manner. Further analyses were done by baseline CRP and MRI status. A higher baseline CRP was associated with significantly better ASAS20 and 40 responses in all tofacitinib treatment groups (*P* ≤ 0.005 in all treatment groups). A positive baseline MRI score also seemed to be associated with a better tofacitinib treatment effect, but this was only significant in the 5 mg twice daily treatment arm. The study was not powered to compare these subgroups.

Subsequent to this, the first phase 3, double-blind RCT of tofacitinib in r-axSpA confirmed significantly greater efficacy at the 5 mg twice daily dosage compared with placebo at week 16 [72]. This study showed that 56% and 40% of patients achieved ASAS20 and ASAS40 responses at week 16, respectively, in the tofacitinib treatment arm compared with 29% and 12% of placebo-treated patients; clinical responses were seen as early as week 2. Significant improvements were also seen in the secondary endpoints, including clinical assessments and patient-reported outcomes assessing disease activity, function, quality of life and mobility. Although not powered to compare subgroups, the descriptive analyses in this study demonstrated significant efficacy in ASAS20/40 response rates with tofacitinib at week 16 when stratified into bDMARD-naïve or TNF-inadequate responder groups. Following patients up to week 48, no new safety signals were identified in the treatment arm, but adverse events were more frequent compared with placebo. When considering safety outcomes of special interest, 2% of patients suffered non-serious herpes zoster, but there were no deaths, malignancies, venous thromboembolism (VTE), major adverse cardiovascular events (MACE) or opportunistic infection. Tofacitinib has subsequently been given a licence for treatment of adults with r-axSpA.

Filgotinib is a more selective JAK1 inhibitor. The TORTUGA phase 2 trial (NCT03117270) showed that filgotinib was efficacious and safe in r-axSpA patients who failed initial management with NSAIDs [73]. This placebo-controlled RCT was the first to assess a selective JAK1 inhibitor in axSpA. Outcomes measured included disease activity, MRI inflammation, clinical features, physical function, health-related quality of life and safety. The primary outcome was the change in ASDAS from baseline to week 12. Results showed disease activity greatly reduced in the filgotinib group by week 12. The mean change in ASDAS was −1.47 (s.d. 1.04) in the filgotinib group compared with −0.57 (s.d. 0.82) in the placebo group. In addition, at week 12 an ASAS20 response was achieved in 76% of the filgotinib group compared with 40% of the placebo group. Significant decreases in the BASDAI score at week 12 were also demonstrated in the filgotinib treatment arm compared with placebo, with decreases from baseline of 2.41 *vs* 1.44, respectively. In addition, patient-reported outcomes identified improvements in symptoms and impact of the disease. Significant improvements in ASQoL were shown, with a decrease of 4.76 in the treatment arm *vs* 2.24 in the placebo arm. The SF-36 scores were also shown to improve by 8.44 in the filgotinib arm and 3.44 in the placebo arm. No new safety signals were identified in this study and filgotinib was well tolerated throughout the 12-week study period.

A post hoc analysis of the TORTUGA trial was carried out to assess the effect of filgotinib on spinal lesions [74]. Total spinal inflammation scores decreased from baseline in the filgotinib group but not in the placebo group. Importantly, posterior spinal element inflammation was also assessed in this analysis and shown to be reduced with filgotinib treatment. Analysis of the MRI scores provided imaging evidence for a reduction of spinal inflammation with filgotinib in regions that are crucial for mobility and function. This study showed filgotinib to be superior to placebo in significantly reducing spinal inflammation, especially in the posterolateral elements, vertebral bodies and facet joints. These structures play a key role in spinal function and mobility, and consequently disease progression. Following the phase 2 study, further phase 3 trials in r-axSpA were planned. Unfortunately, the US Food and Drug Administration (FDA) raised concerns over safety issues related to the 200 mg dose, including the effects of this dose on sperm concentrations and the overall risk–benefit profile of the filgotinib 200 mg dose. At the present time, further clinical development is on hold. A phase 3 program entitled OLINGUITO (NCT05785611) is currently enrolling patients and will involve two phase 3, placebo-controlled, multicentre, double-blind studies, each of which will investigate filgotinib in r-axSpA and nr-axSpA.

Upadacitinib is a more selective JAKi developed and licensed for r-axSpA. The SELECT-AXIS 1 study (NCT03178487) is a double-blind, multicentre, placebo-controlled phase 2/3 trial that assessed the efficacy and safety of upadacitinib in 187 patients with r-axSpA over a 2-year period. This trial consisted of two periods involving bDMARD-naïve patients who had active disease and a poor response or intolerance to NSAIDs. In period 1, oral upadacitinib 15 mg was compared with placebo, with the primary endpoint being an ASAS40 response at week 14. A total of 52% of patients in the upadacitinib group demonstrated an ASAS40 response compared with 26% in the placebo arm (*P* < 0.0003) [75]. Period 2 consisted of patients who completed period 1 and had open-label upadacitinib for a total of 104 weeks. A total of 72% and 85% of patients (in the net reclassification improvement analysis and as-observed analysis, respectively) achieved an ASAS40 at week 64. Secondary endpoints included changes in ASDAS and SPARCC MRI spine score and patient proportions who had ASAS partial remission and BASDAI 50 scores. In the study, no safety signals were seen for serious infection, herpes zoster or VTE.

A phase 3, double-blind, randomized placebo-controlled trial investigated upadacitinib in patients with inadequate response to either IL-17i or TNFi. Significantly more patients achieved the primary endpoint of ASAS40 at week 14 with upadacitinib *vs* placebo (45% *vs* 18%; *P* < 0.0001) [76]. Statistically significant improvements were observed with upadacitinib *vs* placebo for all multiplicity-controlled secondary endpoints (*P* < 0.0001). Thus upadacitinib appears to be a treatment option in biologic-resistant patients. These studies are summarized in Table 2.

**Table 2.** Summary of clinical trial data on the effect of JAKis in r-axSpA and nr-axSpA

Reference	Study type	Arms	Primary endpoint	Results	Data for radiographic change
van der Heijde *et al*. [71]	Phase 2 RCT	Tofacitinib 2 mg, 5 mg and 10 mg BD vs PBO	ASAS20 at week 12	5 mg and 10 mg BD dosing superior to PBO: 10 mg 67.4%, 5 mg 63.0%, PBO 27.3%	Dose response in SPARCC SI joint and spine
Deodhar *et al*. [72]	Phase 3 RCT	Tofacitinib 5 mg BD, PBO	ASAS20 at week 16	5 mg BD dosing superior to PBO: 5 mg BD 56.4%, PBO 29.4%	No data
SELECT-AXIS 1, van der Heijde *et al*. [75]	Phase 2/3 RCT	Upadacitinib 15 mg OD, PBO	ASAS40 at week 14	Upadacitinib superior to PBO: upadacitinib 52%, PBO 26%	Significant improvements in SPARCC MRI spine score
van der Heijde *et al*. [76]	Phase 3	Upadacitinib 15 mg OD, PBO	ASAS40 at week 14	Upadacitinib superior to PBO in bDMARD-inadequate responders: upadacitinib 45%, PBO 18%	Significant improvements in SPARCC MRI spine and SI joint score
SELECT-AXIS 2, Deodhar *et al*. [77]	Phase 3	Upadacitinib 15 mg OD, PBO	ASAS40 at week 14	Upadacitinib superior to PBO: upadacitinib 45%, PBO 23%	Significant improvements in SPARCC MRI spine and SI joint score
TORTUGA, van der Heijde *et al*. [73]	Phase 2	Filgotinib 200 mg, PBO	Reduction in ASDAS at week 12	Filgotinib superior to PBO: Filgotinib −1.47, PBO −0.57	Significant improvements in SPARCC MRI spine and SI joint score
TORTUGA, Maksymowych *et al*. [74]	Phase 2	Filgotinib 200 mg, PBO	Change from baseline to week 12 in CANDEN MRI total spine score	Filgotinib superior to PBO in decreasing spinal inflammation, including posterior elements and facet joints	Significant improvements in MRI spinal inflammation

BD: twice daily; OD: once daily.

Recently, a body of evidence has been developing for the use of JAKis in nr-axSpA. SELECT-AXIS 2 (NCT04169373) is a multicentre, randomized, double-blind, placebo-controlled phase 3 trial aimed at assessing the efficacy and safety of upadacitinib in nr-axSpA [77]. A total of 314 patients were enrolled and had active inflammation, indicated by MRI or an elevated CRP and an inadequate response to NSAIDs; none of the patients had radiographic damage. The proportion of patients with an ASAS40 response at week 14 was the primary endpoint. A total of 295 patients, of which 150 received placebo, were treated for 14 weeks. The ASAS40 response rate at week 14 was significantly higher in upadacitinib-treated patients compared with placebo, with rates of 45% *vs* 23% in the two groups, respectively (*P* < 0.0001; 95% CI 12, 32). The study showed similar rates of adverse events in both groups, 48% and 46% in the treatment and placebo groups, respectively. Only 1% of patients in both groups had a serious infection or herpes zoster episode; neutropenia was observed in 3% of patients in the upadacitinib group but none in the placebo group.

The impact of JAKi use on radiographic progression remains an unmet area of study. TNFis have been shown to be protective, but only in long-term studies. A recent meta-analysis of 24 studies, 18 of which included TNFis, concluded that long-term (>4 years) use of TNFis had a protective effect on radiographic progression [78]. The aforementioned COAST-X trial included patients with nr-axSpA and objective signs of inflammation (on MRI or assessed with CRP) who were naïve to bDMARDs [25]. This study showed that at week 16 in patients receiving ixekizumab, there were significant reductions in erosions and increases in backfill and fat lesions in the SI joints *vs* placebo. However, no such evidence exists for JAKis at present. The SURPASS study (NCT03259074), a phase 3 RCT, investigated radiographic progression with secukinumab and adalimumab and found low radiographic progression with no noticeable difference between both treatment arms over 104 weeks [79].

### JAKis in extra-articular disease

The presence of extra-articular features is an important factor when evaluating treatment options for patients with axSpA. TNF monoclonal antibodies have evidence for preventing recurrent uveitis episodes, with the exception of etanercept [80–82], and have shown superiority to etanercept and secukinumab from observational data [83]. A preference for monoclonal antibody TNF is also seen in IBD, with no efficacy demonstrated for etanercept or secukinumab [84–86]. The absence of head-to-head trials for skin psoriasis in axSpA means that most data are taken from the PsA studies, which show superiority of IL-17 inhibition compared with anti-TNF in achieving skin outcomes [87, 88], thus making IL-17 inhibition preferred for patients with severe skin disease.

Therapeutic effects of JAK inhibition on EAMs in axSpA have been demonstrated but need further research. It is unclear whether JAK has a role in the management of uveitis or an impact on uveitis flare rates, as no clinical trials have been carried out to date. Some JAKis, such as tofacitinib and filgotinib, are licensed for the treatment of ulcerative colitis [89]. In addition, upadacitinib has been shown to increase rates of endoscopic remission in Crohn’s disease patients who had an inadequate response or were intolerant to TNFis [90]. JAKis may also improve plaque psoriasis, as shown in a recent meta-analysis demonstrating the efficacy of tofacitinib in treatment-refractory moderate–severe skin disease. Although not investigated with axSpA, a beneficial effect of filgotinib on psoriasis and enthesitis has been demonstrated in the phase 2 EQUATOR trial (NCT03101670) [91]. A greater proportion of the filgotinib group achieved a 75% reduction in their skin disease compared with placebo at week 16, a 30% treatment difference (*P* < 0.0001). Dosing strategies for JAKis vary according to indication. JAKis are not yet licensed for skin psoriasis in the UK and the dose of other drugs, such as secukinumab, varies. The licensed JAKi doses in IBD also vary and the doses used to treat rheumatic conditions did not show efficacy in the IBD phase 2 clinical trials. The IBD doses are often higher, e.g. upadacitinib is licensed at 30–45 mg in Crohn’s disease but at 15 mg in axSpA. axSpA patients often have concomitant IBD and therefore a multidisciplinary team approach is taken to decide on the optimal dosing for that particular patient, considering which disease domain is driving inflammation at a given point in time. Safety signals, discussed below, have been found with JAKis in certain patient populations and long-term observational data will further delineate whether this risk is found in the higher doses used in IBD.

## Safety concerns and influence of comorbidities

During the initial drug development studies, some specific adverse effects were investigated. There are concerns that JAKis can cause the reactivation of herpes zoster virus [92]. A recent systemic literature review of drugs across the JAKi spectrum showed rates of herpes zoster are increased, with rates of 3.23/100 patient-years [93] compared with 1.6/100 patient-years seen with TNFis [94]. Given the risks with biologics, reactivation of tuberculosis (TB) was also investigated. There are limited data on TB reactivation risk, with most obtained from tofacitinib studies. High background TB prevalence affects the risk of contracting it while on immunosuppression, as with bDMARDs.

During drug development studies for tofacitinib across a range of diseases, there were increases in serum lipid levels and incident cancers observed. For this reason the FDA requested a prospective head-to-head safety trial comparing tofacitinib with TNFis. The ORAL Surveillance study (NCT02092467) was a 4-year non-inferiority safety endpoint trial enrolling patients with RA who were >50 years of age and had at least one cardiovascular risk factor. The study was powered to assess two co-primary endpoints: MACE and cancers. Both co-primary endpoints were not met, with non-inferiority of tofacitinib not being confirmed compared with TNFi. Results published have shown rates of MACE were 3.4% in the tofacitinib group compared with 2.6% in the TNFi group. Cancer rates were also higher, with 4.2% developing malignancies *vs* 2.9% in the TNFi group. This has resulted in an FDA black box warning applied to all JAKis in September 2021 [95]. Given the limited data, it is unclear if this risk is similar across all JAKis or whether this may be related to the relative JAK selectivity seen with different agents. The upadacitinib trial identified two patients who developed lymphoma and one patient who developed non-melanoma skin cancer [96, 97]. The filgotinib trial did not have any incident cancer cases [98]. However, the relatively brief phase 2/3 studies conducted in initial drug development are not powered to identify the safety risk for rare but serious side effects, such as MACE and malignancy. Long-term extension studies are likely to be needed to fully understand the cardiovascular and malignancy risk with the different JAK inhibitors. However, it is important to note that the study population in the ORAL Surveillance study had a diagnosis of RA and were at a higher risk for MACE. These patients represent a different demographic to the axSpA population, who are typically younger at disease onset with lower cardiovascular risk. Although caution and shared decision-making with patients is advised, it is unclear how these safety concerns translate to the axSpA population and additional post-marketing surveillance data will be crucial to address this.

Initial random control testing data for JAKis suggested a possible thromboembolism safety signal, but a recent meta-analysis showed no evidence to support current safety warnings of VTE risk in JAK trial patients [99]. However, in the ORAL Surveillance study, a significantly higher rate of death from pulmonary embolism was seen in patients receiving tofacitinib 10 mg twice daily, which led to a reduction in the tofacitinib dose during the trial.

A subsequent cohort trial examined tofacitinib *vs* TNFis [100]. Two cohorts, one of routine care patients and the other mimicking the inclusion and exclusion criteria of the ORAL Surveillance study were created. The hazard ratio (HR) for the former was 1.01 (95% CI 0.83, 1.23) and the latter was 1.24 (95% CI 0.90, 1.69), thus the conclusion that there was no evidence for tofacitinib increasing MACE risk in the real-world setting but it was associated with a higher risk in RA patients with cardiovascular risk factors.

Another recent population-based cohort study examined JAKi *vs* adalimumab in RA patients, with the primary endpoint being the occurrence of a MACE or VTE event. The HR was 1.0 (95% CI 0.7, 1.5) for MACE and 1.1 (95% CI 0.7, 1.6) for VTE, thus the conclusion that JAKis did not increase these adverse events [101].

Short follow-up duration and small patient numbers limit the evaluation of safety data for JAKis in axSpA. The possibility of a lower theoretical risk of adverse events in axSpA patients compared with RA may be due to these patients being less likely to be treated with glucocorticoids and concomitant methotrexate. As mentioned earlier, the axSpA patient population is more likely to be male and younger than RA populations. However, the greater use of NSAIDs may mean that gastrointestinal perforation may be more common in axSpA patients, initially felt to be a very rare side effect of JAKis but with recent data more reassuring [102]. Safety data at 1 year were reassuring for upadacitinib in the SELECT AXIS 1 extension study [103]. It is also important to note that the axSpA trial populations were not enriched for cardiovascular comorbidities or malignancies, as they were in the RA studies.

## Incorporation into new treatment recommendations and conclusions

With their latest update in 2022, the new ASAS-EULAR recommendations for the management of axSpA have now included JAKis in the treatment algorithm for axSpA. Recommendation 9 emphasizes current practice, which is to use TNFi/IL-17i before considering a JAKi. The recommendation has emphasized this order is largely based on a larger evidence base and drug safety record for TNFis/IL-17is and that the lack of head-to-head trials prevents recommendation based on efficaciousness. Recommendation 12 advises switching to a different mode of action in the event of treatment failure and also highlights a role for JAKis. There is a clear unmet need for new medication in axSpA, particularly for patients who cannot be treated with existing biologic therapies or who have failed to respond to these drugs. This is a lifelong illness and treatments can lose efficacy over time, with a loss of remission occurring in 21% of axSpA patients and flares in 16% of axSpA patients on TNFis [104]. The inclusion of JAKis in the management algorithm provides an additional option for treatment.

There is strong evidence confirming the efficacy of JAKis in managing the signs and symptoms of spinal inflammation in patients with r-axSpA and two drugs are now licensed. Approvals are currently in place for the use of tofacitinib and upadacitinib in patients with r-axSpA and for upadacitinib for nr-axSpA. There are no ongoing trials for tofacitinib and filgotinib in this population. The ASAS-EULAR 2022 recommendations acknowledge that there were no publicly available data for nr-axSpA at the time of writing, precluding it from consideration for recommendation, and advises that longer-term studies are needed in nr-axSpA to establish safety and efficacy data.

New treatment modalities such as JAKis create the opportunity to optimize outcomes for patients. Currently not all axSpA patients can be treated with the existing therapies. Certain comorbidities are contraindications to the currently available biologics, e.g. TNFis in patients with cardiac failure or IL-17 in patients with IBD, so we need additional treatment options beyond NSAIDs. JAKis can provide a solution for these patients. Randomized trials in IBD have also shown positive data, suggesting that these may be helpful in patients with extra-articular features related to their axSpA.

Both TNF and IL-17 inhibition have demonstrable efficacy on axial and peripheral symptoms, with TNFis having an effect on all EAMs. IL-17 inhibition has a particular beneficial effect on skin psoriasis, as mentioned previously. This underscores the importance of the presence of EAMs in choosing a particular bDMARD for a given patient. Long-term experience with TNFis means that this is often the first drug of choice for patients with no EAM, and this is reflected in recent guidelines. Predictors of response are lacking for axSpA treatments, especially with IL-17 inhibition, whereas CRP has been shown to be predictive of response with TNFi. It is still unclear whether switching to a different mechanism of action or cycling within a class is the optimal treatment strategy in the presence of failure of the first bDMARD. The bioavailability and cost-effectiveness of the different biosimilars may be a reason to cycle between TNFis and try at least two of them. Head-to-head trial data are lacking for TNFis, IL-17is and JAKis and the SURPASS study gave negative results for radiographic outcomes comparing TNFis with IL-17is. It is currently difficult to prioritize bDMARDs and JAKis in terms of efficacy and safety. A major unmet need remains the demonstration of prevention of structural progression with the various licensed treatments in axSpA.

Beyond first-line therapeutics, the even greater unmet need in clinics currently relates to patients who are biologic experienced. Additional studies would be helpful to confirm that people with refractory or resistant axSpA may also benefit from JAKi after failure or loss of response with a bDMARD. Data in this population are currently limited to tofacitinib only and represents a minority of patients recruited to the phase 3 trial. Further real-world evidence may help to support their use as second- or third-line therapies.

There has also been a strong note of caution raised by the ORAL Surveillance study. As with any other drug, JAKis do carry risks of side effects. This study suggests that, particularly for older patients with high baseline cardiovascular risk, other therapies may be a better option. In the shorter-term RCTs, a number of manageable safety signals were identified, including the risk of herpes zoster, which seems to be specific to this mode of action. Shingles vaccines are recommended prior to starting JAKis in all indications. While caution and careful patient counselling are recommended, it seems likely that the absolute risk of adverse events may be lower in a younger axSpA population. Further long-term extension studies are necessary to accurately identify the risk of cardiovascular effects and malignancy rates in the axSpA population.

At the present time, JAKis provide a novel treatment for axSpA, the first effective oral agent for inflammatory spinal disease. While first-line use is likely to be limited by current safety concerns, they address some of the unmet needs in axSpA for those patients in whom existing treatment options have failed or are contraindicated.

## Data Availability

No new data were generated or analysed in support of this article.
